# Supplementary material for: The effects of antibiotics and illness on gut microbial composition in the fawn-footed mosaic-tailed rat (Melomys cervinipes)
Source: PLoS One. 2023 Feb 24;18(2):e0281533. doi: 10.1371/journal.pone.0281533 (PMC9956021; doi:10.1371/journal.pone.0281533)
Supplement: S1 Table — Different bacterial group abundances and the effects of treatment, sex, birth and their interactions. The * refers to results that are significant at the α = 0.05 level, and significant results are discussed in the main text. (DOCX) [file pone.0281533.s001.docx]

**S1 Table.** **Output of rank-based non-parametric analyses for longitudinal data models of different bacterial group abundances**. The effects of treatment, sex, birth and their interactions. Cohen’s *d* effect sizes and confidence intervals provided. The * refers to results that are significant at the α = 0.05 level, and significant results are discussed in the main text.

| **Anova-Type Statistics (ATS)** | | | | |
| --- | --- | --- | --- | --- |
| **Model** | **Abundance.Bacteria ∼ Treatment + Sex+Birth, subject = factor (ID)** | | | |
|  | **Statistic** | **df** | **p-value** | **Cohen’s *d* [+CIs]** |
| **Sex** | 0.57 | 1 | 0.450 | - 0.24 [-0.98, 0.52] |
| **Birth** | 1.21 | 1 | 0.272 | 0.47 [-0.29, 1.23] |
| **Treatment** | 41.17 | 1 | < 0.001 * | 1.52 [0.66, 2.35] |
| **Sex * Birth** | 0.03 | 1 | 0.874 | - |
| **Sex * Treatment** | 0.35 | 1 | 0.557 | - |
| **Birth * Treatment** | 0.00 | 1 | 0.945 | - |
| **Sex * Birth * Treatment** | 1.96 | 1 | 0.161 | - |
|  |  |  |  |  |
| **Model.1** | **PC_Phylum1 ∼ Treatment + Sex+Birth, subject = factor (ID)** | | | |
|  | **Statistic** | **df** | **p-value** | **Cohen’s *d* [+CIs]** |
| **Sex** | 0.15 | 1 | 0.699 | 0.49 [-0.28, 1.24] |
| **Birth** | 0.39 | 1 | 0.533 | -0.14 [-0.88, 0.62] |
| **Treatment** | 0.13 | 1 | 0.715 | 0.61 [-0.15, 1.37] |
| **Sex * Birth** | 1.49 | 1 | 0.222 | - |
| **Sex * Treatment** | 1.96 | 1 | 0.161 | - |
| **Birth * Treatment** | 0.04 | 1 | 0.851 | - |
| **Sex * Birth * Treatment** | 0.60 | 1 | 0.437 | - |
|  |  |  |  |  |
| **Model.2** | **PC_Phylum2 ∼ Treatment + Sex+Birth, subject = factor (ID)** | | | |
|  | **Statistic** | **df** | **p-value** | **Cohen’s *d* [+CIs]** |
| **Sex** | 0.70 | 1 | 0.403 | -0.37 [-1.12, 0.39] |
| **Birth** | 0.60 | 1 | 0.438 | -0.42 [-1.17, 0.34] |
| **Treatment** | 9.51 | 1 | 0.002 * | -0.85 [-1.62, 0.06] |
| **Sex * Birth** | 0.35 | 1 | 0.552 | - |
| **Sex * Treatment** | 0.01 | 1 | 0.939 | - |
| **Birth * Treatment** | 1.78 | 1 | 0.182 | - |
| **Sex * Birth * Treatment** | 1.03 | 1 | 0.309 | - |
|  |  |  |  |  |
| **Model.3** | **Pseudomonadata ∼ Treatment + Sex+Birth, subject = factor (ID)** | | | |
|  | **Statistic** | **df** | **p-value** | **Cohen’s *d* [+CIs]** |
| **Sex** | 2.29 | 1 | 0.130 | -0.70 [-1.47, 0.08] |
| **Birth** | 1.12 | 1 | 0.290 | 0.25 [-0.51, 1.00] |
| **Treatment** | 18.62 | 1 | 0.002 * | -0.79 [-1.56, -0.02] |
| **Sex * Birth** | 0.60 | 1 | 0.439 | - |
| **Sex * Treatment** | 3.69 | 1 | 0.055 | - |
| **Birth * Treatment** | 14.60 | 1 | < 0.001 | - |
| **Sex * Birth * Treatment** | 3.32 | 1 | 0.069 | - |
|  |  |  |  |  |
| **Model.4** | **Bacillota ∼ Treatment + Sex+Birth, subject = factor (ID)** | | | |
|  | **Statistic** | **df** | **p-value** | **Cohen’s *d* [+CIs]** |
| **Sex** | 0.74 | 1 | 0.388 | 0.37 [-0.39, 1.12] |
| **Birth** | 0.65 | 1 | 0.422 | 0.42 [-0.34, 1.17] |
| **Treatment** | 10.04 | 1 | 0.002 * | 0.93 [0.14, 1.71] |
| **Sex * Birth** | 0.32 | 1 | 0.570 | - |
| **Sex * Treatment** | 0.00 | 1 | 0.965 | - |
| **Birth * Treatment** | 1.78 | 1 | 0.180 | - |
| **Sex * Birth * Treatment** | 0.77 | 1 | 0.381 | - |
|  |  |  |  |  |
| **Model.5** | **PC_Class1 ∼ Treatment + Sex+Birth, subject = factor (ID)** | | | |
|  | **Statistic** | **df** | **p-value** | **Cohen’s *d* [+CIs]** |
| **Sex** | 0.15 | 1 | 0.696 | 0.47 [-0.30, 1.22] |
| **Birth** | 0.46 | 1 | 0.500 | -0.16 [-0.90, 0.60] |
| **Treatment** | 0.11 | 1 | 0.744 | 0.55 [-0.21, 1.30] |
| **Sex * Birth** | 0.94 | 1 | 0.333 | - |
| **Sex * Treatment** | 0.98 | 1 | 0.323 | - |
| **Birth * Treatment** | 0.06 | 1 | 0.801 | - |
| **Sex * Birth * Treatment** | 0.29 | 1 | 0.592 | - |
| **Model.6** | **PC_Class2 ∼ Treatment + Sex+Birth, subject = factor (ID)** | | | |
|  | **Statistic** | **df** | **p-value** | **Cohen’s *d* [+CIs]** |
| **Sex** | 0.91 | 1 | 0.339 | -0.45 [-1.21, 0.31] |
| **Birth** | 0.82 | 1 | 0.366 | -0.50 [-1.26, 0.26] |
| **Treatment** | 10.26 | 1 | 0.001 * | -1.05 [-1.83, -0.25] |
| **Sex * Birth** | 0.41 | 1 | 0.521 | - |
| **Sex * Treatment** | 0.03 | 1 | 0.864 | - |
| **Birth * Treatment** | 0.33 | 1 | 0.567 | - |
| **Sex * Birth * Treatment** | 0.03 | 1 | 0.864 | - |
|  |  |  |  |  |
| **Model.7** | **Bacilli ∼ Treatment + Sex+Birth, subject = factor (ID)** | | | |
|  | **Statistic** | **df** | **p-value** | **Cohen’s *d* [+CIs]** |
| **Sex** | 2.41 | 1 | 0.121 | -0.52 [-1.27, 0.25] |
| **Birth** | 1.67 | 1 | 0.196 | 0.12 [-0.63, 0.86] |
| **Treatment** | 6.96 | 1 | 0.008 * | 0.97 [0.18, 1.75] |
| **Sex * Birth** | 0.51 | 1 | 0.477 | - |
| **Sex * Treatment** | 0.12 | 1 | 0.726 | - |
| **Birth * Treatment** | 2.31 | 1 | 0.128 | - |
| **Sex * Birth * Treatment** | 1.42 | 1 | 0.234 | - |
|  |  |  |  |  |
| **Model.8** | **Clostridia ∼ Treatment + Sex+Birth, subject = factor (ID)** | | | |
|  | **Statistic** | **df** | **p-value** | **Cohen’s *d* [+CIs]** |
| **Sex** | 1.26 | 1 | 0.261 | 0.63 [-0.14, 1.40] |
| **Birth** | 0.51 | 1 | 0.476 | 0.46 [-0.30, 1.21] |
| **Treatment** | 8.49 | 1 | 0.004 * | 0.95 [0.16, 1.73] |
| **Sex * Birth** | 0.72 | 1 | 0.395 | - |
| **Sex * Treatment** | 0.20 | 1 | 0.654 | - |
| **Birth * Treatment** | 0.45 | 1 | 0.501 | - |
| **Sex * Birth * Treatment** | 0.55 | 1 | 0.460 | - |
|  |  |  |  |  |
| **Model.9** | **Negativicutes ∼ Treatment + Sex+Birth, subject = factor (ID)** | | | |
|  | **Statistic** | **df** | **p-value** | **Cohen’s *d* [+CIs]** |
| **Sex** | 0.48 | 1 | 0.490 | 0.66 [-0.12, 1.42] |
| **Birth** | 0.26 | 1 | 0.610 | -0.24 [-0.99, 0.51] |
| **Treatment** | 9.68 | 1 | 0.002 * | -1.00 [-1.78, -0.21] |
| **Sex * Birth** | 0.01 | 1 | 0.912 | - |
| **Sex * Treatment** | 2.30 | 1 | 0.130 | - |
| **Birth * Treatment** | 1.25 | 1 | 0.264 | - |
| **Sex * Birth * Treatment** | 0.10 | 1 | 0.750 | - |
|  |  |  |  |  |
| **Model.10** | **PC_Order1 ∼ Treatment + Sex+Birth, subject = factor (ID)** | | | |
|  | **Statistic** | **df** | **p-value** | **Cohen’s *d* [+CIs]** |
| **Sex** | 0.14 | 1 | 0.704 | 0.33 [-0.42, 1.08] |
| **Birth** | 0.43 | 1 | 0.510 | -0.13 [-0.88, 0.62] |
| **Treatment** | 21.90 | 1 | < 0.001 * | 1.09 [0.28, 1.87] |
| **Sex * Birth** | 0.32 | 1 | 0.569 | - |
| **Sex * Treatment** | 0.08 | 1 | 0.777 | - |
| **Birth * Treatment** | 0.41 | 1 | 0.523 | - |
| **Sex * Birth * Treatment** | 0.85 | 1 | 0.357 | - |
|  |  |  |  |  |
| **Model.11** | **PC_Order2 ∼ Treatment + Sex+Birth, subject = factor (ID)** | | | |
|  | **Statistic** | **df** | **p-value** | **Cohen’s *d* [+CIs]** |
| **Sex** | 2.14 | 1 | 0.144 | -0.38 [-1.13, 0.38] |
| **Birth** | 0.63 | 1 | 0.427 | 0.04 [-0.70, 0.79] |
| **Treatment** | 46.05 | 1 | < 0.001 * | 2.80 [1.73, 3.85] |
| **Sex * Birth** | 1.21 | 1 | 0.271 | - |
| **Sex * Treatment** | 1.91 | 1 | 0.167 | - |
| **Birth * Treatment** | 0.88 | 1 | 0.348 | - |
| **Sex * Birth * Treatment** | 0.85 | 1 | 0.357 | - |
|  |  |  |  |  |
| **Model.12** | **PC_Order3 ∼ Treatment + Sex+Birth, subject = factor (ID)** | | | |
|  | **Statistic** | **df** | **p-value** | **Cohen’s *d* [+CIs]** |
| **Sex** | 0.63 | 1 | 0.426 | 0.07 [-0.68, 0.82] |
| **Birth** | 0.97 | 1 | 0.325 | 0.66 [-0.11, 1.43] |
| **Treatment** | 0.05 | 1 | 0.821 | 0.05 [-0.69, 0.79] |
| **Sex * Birth** | 0.63 | 1 | 0.426 | - |
| **Sex * Treatment** | 0.17 | 1 | 0.678 | - |
| **Birth * Treatment** | 0.26 | 1 | 0.610 | - |
| **Sex * Birth * Treatment** | 0.66 | 1 | 0.417 | - |
|  |  |  |  |  |
| **Model.13** | **PC_Order4 ∼ Treatment + Sex+Birth, subject = factor (ID)** | | | |
|  | **Statistic** | **df** | **p-value** | **Cohen’s *d* [+CIs]** |
| **Sex** | 0.53 | 1 | 0.465 | -0.37 [-1.12, 0.39] |
| **Birth** | 0.06 | 1 | 0.808 | -0.26 [-1.01, 0.49] |
| **Treatment** | 0.05 | 1 | 0.818 | 0.02 [-0.54, 0.94] |
| **Sex * Birth** | 0.00 | 1 | 0.961 | - |
| **Sex * Treatment** | 1.07 | 1 | 0.300 | - |
| **Birth * Treatment** | 2.60 | 1 | 0.107 | - |
| **Sex * Birth * Treatment** | 5.84 | 1 | 0.016 * | - |
|  |  |  |  |  |
| **Model.14** | **Fusobacteriales ∼ Treatment + Sex+Birth, subject = factor (ID)** | | | |
|  | **Statistic** | **df** | **p-value** | **Cohen’s *d* [+CIs]** |
| **Sex** | 0.00 | 1 | 0.948 | -0.38 [-1.13, 0.38] |
| **Birth** | 1.05 | 1 | 0.306 | -0.55 [-1.30, 0.22] |
| **Treatment** | 5.62 | 1 | 0.018 * | -0.45 [-1.19, 0.31] |
| **Sex * Birth** | 0.03 | 1 | 0.861 | - |
| **Sex * Treatment** | 2.25 | 1 | 0.133 | - |
| **Birth * Treatment** | 0.16 | 1 | 0.688 | - |
| **Sex * Birth * Treatment** | 1.17 | 1 | 0.280 | - |
|  |  |  |  |  |
| **Model.15** | **Enterobacterales ∼ Treatment + Sex+Birth, subject = factor (ID)** | | | |
|  | **Statistic** | **df** | **p-value** | **Cohen’s *d* [+CIs]** |
| **Sex** | 0.01 | 1 | 0.910 | -0.53 [-1.29, 0.24] |
| **Birth** | 1.26 | 1 | 0.262 | 0.20 [-0.56, 0.95] |
| **Treatment** | 5.20 | 1 | 0.023 * | -0.49 [-1.24, 0.27] |
| **Sex * Birth** | 1.64 | 1 | 0.200 | - |
| **Sex * Treatment** | 0.28 | 1 | 0.599 | - |
| **Birth * Treatment** | 0.00 | 1 | 0.953 | - |
| **Sex * Birth * Treatment** | 0.05 | 1 | 0.815 | - |
|  |  |  |  |  |
| **Model.16** | **Eubacteriales ∼ Treatment + Sex+Birth, subject = factor (ID)** | | | |
|  | **Statistic** | **df** | **p-value** | **Cohen’s *d* [+CIs]** |
| **Sex** | 1.92 | 1 | 0.166 | 0.22 [-0.53, 0.97] |
| **Birth** | 1.31 | 1 | 0.252 | 0.10 [-0.65, 0.85] |
| **Treatment** | 210.55 | 1 | < 0.001 * | -5.90 [-7.65, -4.13] |
| **Sex * Birth** | 1.31 | 1 | 0.252 | - |
| **Sex * Treatment** | 1.39 | 1 | 0.238 | - |
| **Birth * Treatment** | 2.16 | 1 | 0.142 | - |
| **Sex * Birth * Treatment** | 0.62 | 1 | 0.432 | - |
|  |  |  |  |  |
| **Model.16** | **Selenomonadales ∼ Treatment + Sex+Birth, subject = factor (ID)** | | | |
|  | **Statistic** | **df** | **p-value** | **Cohen’s *d* [+CIs]** |
| **Sex** | 0.48 | 1 | 0.490 | 0.66 [-0.12, 1.42] |
| **Birth** | 0.26 | 1 | 0.610 | -0.24 [-0.99, 0.51] |
| **Treatment** | 9.68 | 1 | 0.002 * | -1.00 [-1.78, -0.21] |
| **Sex * Birth** | 0.01 | 1 | 0.912 | - |
| **Sex * Treatment** | 2.30 | 1 | 0.130 | - |
| **Birth * Treatment** | 1.25 | 1 | 0.264 | - |
| **Sex * Birth * Treatment** | 0.10 | 1 | 0.750 | - |
|  |  |  |  |  |
| **Model.17** | **Erysipelotrichales ∼ Treatment + Sex+Birth, subject = factor (ID)** | | | |
|  | **Statistic** | **df** | **p-value** | **Cohen’s *d* [+CIs]** |
| **Sex** | 0.11 | 1 | 0.736 | -0.02 [-0.77, 0.73] |
| **Birth** | 1.00 | 1 | 0.316 | 0.34 [-0.42, 1.09] |
| **Treatment** | 10.27 | 1 | 0.001 * | 1.14 [0.32, 1.93] |
| **Sex * Birth** | 0.70 | 1 | 0.402 | - |
| **Sex * Treatment** | 2.66 | 1 | 0.103 | - |
| **Birth * Treatment** | 4.70 | 1 | 0.030 * | - |
| **Sex * Birth * Treatment** | 0.35 | 1 | 0.553 | - |
|  |  |  |  |  |
| **Model.18** | **Rhodospirillales ∼ Treatment + Sex+Birth, subject = factor (ID)** | | | |
|  | **Statistic** | **df** | **p-value** | **Cohen’s *d* [+CIs]** |
| **Sex** | 0.11 | 1 | 0.736 | -0.04 [-0.79, 0.71] |
| **Birth** | 0.39 | 1 | 0.534 | -0.08 [-0.82, 0.67] |
| **Treatment** | 0.01 | 1 | 0.922 | -0.53 [-1.28, 0.23] |
| **Sex * Birth** | 0.39 | 1 | 0.534 | - |
| **Sex * Treatment** | 0.18 | 1 | 0.673 | - |
| **Birth * Treatment** | 0.63 | 1 | 0.429 | - |
| **Sex * Birth * Treatment** | 3.06 | 1 | 0.080 | - |
|  |  |  |  |  |
| **Model.19** | **PC_Family1 ∼ Treatment + Sex+Birth, subject = factor (ID)** | | | |
|  | **Statistic** | **df** | **p-value** | **Cohen’s *d* [+CIs]** |
| **Sex** | 4.00 | 1 | 0.046 * | -0.42 [-1.17, 0.35] |
| **Birth** | 0.07 | 1 | 0.792 | 0.13 [-0.62, 0.88] |
| **Treatment** | 34.41 | 1 | < 0.001 * | 2.48 [1.46, 3.46] |
| **Sex * Birth** | 3.49 | 1 | 0.062 | - |
| **Sex * Treatment** | 0.02 | 1 | 0.888 | - |
| **Birth * Treatment** | 0.00 | 1 | 0.987 | - |
| **Sex * Birth * Treatment** | 1.35 | 1 | 0.246 | - |
|  |  |  |  |  |
| **Model.20** | **PC_Family2 ∼ Treatment + Sex+Birth, subject = factor (ID)** | | | |
|  | **Statistic** | **df** | **p-value** | **Cohen’s *d* [+CIs]** |
| **Sex** | 6.11 | 1 | 0.013 * | -0.85 [-1.62, -0.06] |
| **Birth** | 0.71 | 1 | 0.399 | 0.03 [-0.71, 0.78] |
| **Treatment** | 1.91 | 1 | 0.168 | 0.16 [-0.58, 0.90] |
| **Sex * Birth** | 0.00 | 1 | 0.955 | - |
| **Sex * Treatment** | 1.61 | 1 | 0.204 | - |
| **Birth * Treatment** | 0.01 | 1 | 0.912 | - |
| **Sex * Birth * Treatment** | 0.20 | 1 | 0.659 | - |
|  |  |  |  |  |
| **Model.21** | **PC_Family3 ∼ Treatment + Sex+Birth, subject = factor (ID)** | | | |
|  | **Statistic** | **df** | **p-value** | **Cohen’s *d* [+CIs]** |
| **Sex** | 1.29 | 1 | 0.256 | 0.28 [-0.47, 1.03] |
| **Birth** | 1.41 | 1 | 0.235 | 0.49 [-0.27, 1.25] |
| **Treatment** | 0.10 | 1 | 0.754 | 0.02 [-0.72, 0.76] |
| **Sex * Birth** | 0.92 | 1 | 0.336 | - |
| **Sex * Treatment** | 0.00 | 1 | 0.977 | - |
| **Birth * Treatment** | 0.24 | 1 | 0.621 | - |
| **Sex * Birth * Treatment** | 1.98 | 1 | 0.160 | - |
|  |  |  |  |  |
| **Model.22** | **PC_Family4 ∼ Treatment + Sex+Birth, subject = factor (ID)** | | | |
|  | **Statistic** | **df** | **p-value** | **Cohen’s *d* [+CIs]** |
| **Sex** | 0.91 | 1 | 0.340 | -0.62 [-1.38, 0.15] |
| **Birth** | 0.68 | 1 | 0.408 | -0.57 [-1.33, 0.20] |
| **Treatment** | 4.36 | 1 | 0.037 * | -0.49 [-1.24, 0.27] |
| **Sex * Birth** | 0.49 | 1 | 0.484 | - |
| **Sex * Treatment** | 0.15 | 1 | 0.696 | - |
| **Birth * Treatment** | 0.19 | 1 | 0.663 | - |
| **Sex * Birth * Treatment** | 4.18 | 1 | 0.041 * | - |
|  |  |  |  |  |
| **Model.23** | **PC_Family5 ∼ Treatment + Sex+Birth, subject = factor (ID)** | | | |
|  | **Statistic** | **df** | **p-value** | **Cohen’s *d* [+CIs]** |
| **Sex** | 1.16 | 1 | 0.280 | 0.38 [-0.38, 1.13] |
| **Birth** | 0.03 | 1 | 0.871 | -0.25 [-1.00, 0.50] |
| **Treatment** | 1.55 | 1 | 0.214 | 0.27 [-0.48, 1.01] |
| **Sex * Birth** | 0.61 | 1 | 0.434 | - |
| **Sex * Treatment** | 1.17 | 1 | 0.280 | - |
| **Birth * Treatment** | 3.79 | 1 | 0.052 | - |
| **Sex * Birth * Treatment** | 0.24 | 1 | 0.627 | - |
|  |  |  |  |  |
| **Model.24** | **PC_Family6 ∼ Treatment + Sex+Birth, subject = factor (ID)** | | | |
|  | **Statistic** | **df** | **p-value** | **Cohen’s *d* [+CIs]** |
| **Sex** | 0.04 | 1 | 0.845 | -0.11 [-0.86, 0.64] |
| **Birth** | 0.76 | 1 | 0.383 | -0.06 [-0.81, 0.69] |
| **Treatment** | 0.99 | 1 | 0.319 | 0.39 [-0.37, 1.13] |
| **Sex * Birth** | 18.40 | 1 | < 0.001 * | - |
| **Sex * Treatment** | 1.84 | 1 | 0.175 | - |
| **Birth * Treatment** | 0.02 | 1 | 0.880 | - |
| **Sex * Birth * Treatment** | 0.02 | 1 | 0.895 | - |
|  |  |  |  |  |
| **Model.25** | **Eubacteriaceae ∼ Treatment + Sex+Birth, subject = factor (ID)** | | | |
|  | **Statistic** | **df** | **p-value** | **Cohen’s *d* [+CIs]** |
| **Sex** | 0.01 | 1 | 0.922 | 0.42 [-0.34, 1.17] |
| **Birth** | 0.15 | 1 | 0.696 | -0.12 [-0.86, 0.63] |
| **Treatment** | 58.76 | 1 | < 0.001 * | 1.35 [0.52, 2.17] |
| **Sex * Birth** | 1.38 | 1 | 0.241 | - |
| **Sex * Treatment** | 1.28 | 1 | 0.257 | - |
| **Birth * Treatment** | 0.48 | 1 | 0.489 | - |
| **Sex * Birth * Treatment** | 0.43 | 1 | 0.514 | - |
|  |  |  |  |  |
| **Model.26** | **Muribaculaceae ∼ Treatment + Sex+Birth, subject = factor (ID)** | | | |
|  | **Statistic** | **df** | **p-value** | **Cohen’s *d* [+CIs]** |
| **Sex** | 4.29 | 1 | 0.038 * | -0.42 [-1.18, 0.34] |
| **Birth** | 0.27 | 1 | 0.604 | 0.09 [-0.66, 0.84] |
| **Treatment** | 48.02 | 1 | < 0.001 * | 2.28 [1.30, 3.23] |
| **Sex * Birth** | 3.83 | 1 | 0.050 | - |
| **Sex * Treatment** | 0.03 | 1 | 0.871 | - |
| **Birth * Treatment** | 0.01 | 1 | 0.943 | - |
| **Sex * Birth * Treatment** | 1.13 | 1 | 0.287 | - |
|  |  |  |  |  |
| **Model.27** | **Peptostreptococcaceae ∼ Treatment + Sex+Birth, subject = factor (ID)** | | | |
|  | **Statistic** | **df** | **p-value** | **Cohen’s *d* [+CIs]** |
| **Sex** | 6.94 | 1 | 0.008 * | 0.46 [-0.30, 1.22] |
| **Birth** | 0.56 | 1 | 0.444 | 0.01 [-0.74, 0.76] |
| **Treatment** | 39.04 | 1 | < 0.001 * | -2.31 [-3.26, -1.33] |
| **Sex * Birth** | 0.23 | 1 | 0.630 | - |
| **Sex * Treatment** | 0.53 | 1 | 0.465 | - |
| **Birth * Treatment** | 0.80 | 1 | 0.372 | - |
| **Sex * Birth * Treatment** | 0.01 | 1 | 0.935 | - |
|  |  |  |  |  |
| **Model.28** | **Tannerellacaeae ∼ Treatment + Sex+Birth, subject = factor (ID)** | | | |
|  | **Statistic** | **df** | **p-value** | **Cohen’s *d* [+CIs]** |
| **Sex** | 3.04 | 1 | 0.081 | 0.70 [-0.08, 1.46] |
| **Birth** | 0.01 | 1 | 0.942 | -0.13 [-0.88, 0.62] |
| **Treatment** | 19.17 | 1 | < 0.001 * | -1.56 [-2.40, -0.70] |
| **Sex * Birth** | 0.71 | 1 | 0.399 | - |
| **Sex * Treatment** | 6.48 | 1 | 0.011 * | - |
| **Birth * Treatment** | 1.01 | 1 | 0.316 | - |
| **Sex * Birth * Treatment** | 0.00 | 1 | 0.954 | - |
|  |  |  |  |  |
| **Model.29** | **Oscillospiraceae ∼ Treatment + Sex+Birth, subject = factor (ID)** | | | |
|  | **Statistic** | **df** | **p-value** | **Cohen’s *d* [+CIs]** |
| **Sex** | 0.00 | 1 | 0.978 | -0.14 [-0.89, 0.61] |
| **Birth** | 0.15 | 1 | 0.702 | 0.37 [-0.38, 1.13] |
| **Treatment** | 9.93 | 1 | 0.002 * | 0.86 [0.08, 1.63] |
| **Sex * Birth** | 0.22 | 1 | 0.636 | - |
| **Sex * Treatment** | 0.00 | 1 | 0.945 | - |
| **Birth * Treatment** | 0.49 | 1 | 0.483 | - |
| **Sex * Birth * Treatment** | 0.63 | 1 | 0.427 | - |
|  |  |  |  |  |
| **Model.30** | **Rikenellaceae ∼ Treatment + Sex+Birth, subject = factor (ID)** | | | |
|  | **Statistic** | **df** | **p-value** | **Cohen’s *d* [+CIs]** |
| **Sex** | 15.25 | 1 | < 0.001 * | 0.92 [0.12, 1.70] |
| **Birth** | 2.71 | 1 | 0.099 | -0.59 [-1.35, 0.18] |
| **Treatment** | 2.95 | 1 | 0.086 | -0.75 [-1.51, 0.02] |
| **Sex * Birth** | 0.95 | 1 | 0.329 | - |
| **Sex * Treatment** | 1.09 | 1 | 0.296 | - |
| **Birth * Treatment** | 0.27 | 1 | 0.601 | - |
| **Sex * Birth * Treatment** | 0.36 | 1 | 0.550 | - |
|  |  |  |  |  |
| **Model.31** | **Rhodospirillales (uncultured) ∼ Treatment + Sex+Birth, subject = factor (ID)** | | | |
|  | **Statistic** | **df** | **p-value** | **Cohen’s *d* [+CIs]** |
| **Sex** | 0.11 | 1 | 0.736 | -0.04 [-0.79, 0.71] |
| **Birth** | 0.39 | 1 | 0.534 | -0.08 [-0.82, 0.67] |
| **Treatment** | 0.01 | 1 | 0.922 | -0.53 [-1.28, 0.23] |
| **Sex * Birth** | 0.39 | 1 | 0.534 | - |
| **Sex * Treatment** | 0.18 | 1 | 0.673 | - |
| **Birth * Treatment** | 0.63 | 1 | 0.429 | - |
| **Sex * Birth * Treatment** | 3.06 | 1 | 0.080 | - |
