# Supplementary material for: The effects of antibiotics and illness on gut microbial composition in the fawn-footed mosaic-tailed rat (Melomys cervinipes)
Source: PLoS One. 2023 Feb 24;18(2):e0281533. doi: 10.1371/journal.pone.0281533 (PMC9956021; doi:10.1371/journal.pone.0281533)
Supplement: S2 Table — Generated from the abundance of Bacteria in fawn-footed mosaic-tailed rats (Melomys cervinipes) in two treatments (TREATMENT and POST-TREATMENT). (DOCX) [file pone.0281533.s002.docx]

**S2 Table.** **Outputs of principle components analyses**. Generated from the abundance of Bacteria in fawn-footed mosaic-tailed rats (*Melomys cervinipes*) in two treatments (TREATMENT and POST-TREATMENT).

| **PC** | **Eigen Value** | **Proportion of Variance** | **Variable** | **Loadings** | **Contribution (%)** |
| --- | --- | --- | --- | --- | --- |
| PC_Phylum1 | 2.67 | 0.44 | Bacteroidota | 0.81 | 24.79 |
|  |  |  | Cyanobacteria | 0.03 | 0.04 |
|  |  |  | Bacillota | 0.37 | 5.27 |
|  |  |  | Fusobacteriota | -0.78 | 22.81 |
|  |  |  | Pseudomonadata | -0.66 | 16.52 |
|  |  |  | Verrucomicrobiota | -0.90 | 30.58 |
| PC_Phylum2 | 1.31 | 0.22 | Bacteroidota | 0.40 | 12.43 |
|  |  |  | Cyanobacteria | 0.61 | 28.20 |
|  |  |  | Bacillota | -0.85 | 54.49 |
|  |  |  | Fusobacteriota | -0.19 | 2.80 |
|  |  |  | Pseudomonadata | 0.12 | 1.14 |
|  |  |  | Verrucomicrobiota | 0.11 | 0.94 |
| PC_Class1 | 2.73 | 0.34 | Alphaproteobacteria | 0.01 | 0.01 |
|  |  |  | Bacilli | 0.31 | 3.51 |
|  |  |  | Bacteroidia | 0.79 | 22.81 |
|  |  |  | Clostridia | 0.34 | 4.29 |
|  |  |  | Fusobacteriia | -0.76 | 21.14 |
|  |  |  | Gammaproteobacteria | -0.66 | 15.80 |
|  |  |  | Negativicutes | -0.22 | 1.77 |
|  |  |  | Verrucomicrobiae | -0.91 | 30.66 |
| PC_Class2 | 2.73 | 0.34 | Alphaproteobacteria | -0.64 | 19.26 |
|  |  |  | Bacilli | 0.63 | 18.69 |
|  |  |  | Bacteroidia | -0.51 | 12.53 |
|  |  |  | Clostridia | 0.65 | 20.29 |
|  |  |  | Fusobacteriia | 0.14 | 0.87 |
|  |  |  | Gammaproteobacteria | 0.21 | 2.18 |
|  |  |  | Negativicutes | -0.74 | 25.82 |
|  |  |  | Verrucomicrobiae | -0.09 | 0.35 |
| PC_Order1 | 2.98 | 0.30 | Bacteroidales | 0.77 | 20.05 |
|  |  |  | Enterobacterales | -0.65 | 14.25 |
|  |  |  | Erysipelotrichales | 0.48 | 7.70 |
|  |  |  | Eubacteriales | -0.56 | 10.48 |
|  |  |  | Fusobacteriales | -0.74 | 18.58 |
|  |  |  | Gastranaerophilales | 0.01 | 0.00 |
|  |  |  | Lactobacillales | -0.32 | 3.51 |
|  |  |  | Rhodospirillales | 0.00 | 0.00 |
|  |  |  | Selenomonadales | -0.19 | 1.25 |
|  |  |  | Verrucomicrobiales | -0.85 | 24.18 |
| PC_Order2 | 2.02 | 0.20 | Bacteroidales | -0.48 | 11.33 |
|  |  |  | Enterobacterales | 0.33 | 5.44 |
|  |  |  | Erysipelotrichales | 0.55 | 14.91 |
|  |  |  | Eubacteriales | -0.56 | 15.57 |
|  |  |  | Fusobacteriales | 0.19 | 1.78 |
|  |  |  | Gastranaerophilales | -0.44 | 9.56 |
|  |  |  | Lactobacillales | -0.21 | 2.26 |
|  |  |  | Rhodospirillales | -0.54 | 14.54 |
|  |  |  | Selenomonadales | -0.70 | 24.42 |
|  |  |  | Verrucomicrobiales | 0.06 | 0.18 |
| PC_Order3 | 1.28 | 0.13 | Bacteroidales | 0.23 | 3.97 |
|  |  |  | Enterobacterales | -0.00 | 0.00 |
|  |  |  | Erysipelotrichales | -0.44 | 15.26 |
|  |  |  | Eubacteriales | -0.08 | 0.50 |
|  |  |  | Fusobacteriales | 0.07 | 0.42 |
|  |  |  | Gastranaerophilales | -0.77 | 46.97 |
|  |  |  | Lactobacillales | -0.07 | 0.37 |
|  |  |  | Rhodospirillales | 0.55 | 23.71 |
|  |  |  | Selenomonadales | -0.33 | 8.65 |
|  |  |  | Verrucomicrobiales | 0.04 | 0.14 |
|  |  |  |  |  |  |
|  |  |  |  |  |  |
|  |  |  |  |  |  |
| PC_Order4 | 1.06 | 0.11 | Bacteroidales | -0.06 | 0.29 |
|  |  |  | Enterobacterales | -0.08 | 0.59 |
|  |  |  | Erysipelotrichales | 0.14 | 1.79 |
|  |  |  | Eubacteriales | -0.21 | 4.09 |
|  |  |  | Fusobacteriales | 0.15 | 2.09 |
|  |  |  | Gastranaerophilales | 0.05 | 0.27 |
|  |  |  | Lactobacillales | -0.82 | 62.69 |
|  |  |  | Rhodospirillales | 0.25 | 5.89 |
|  |  |  | Selenomonadales | 0.36 | 12.48 |
|  |  |  | Verrucomicrobiales | 0.32 | 9.83 |
| PC_Class1 | 5.01 | 0.29 | Akkermansiaceae | -0.35 | 2.49 |
|  |  |  | Bacteroidaceae | -0.68 | 9.12 |
|  |  |  | Clostridiales vadinBB60 group | 0.47 | 4.45 |
|  |  |  | Enterobacteriaceae | -0.12 | 0.26 |
|  |  |  | Erysipelotrichaceae | 0.56 | 6.62 |
|  |  |  | Eubacteriaceae | 0.64 | 8.05 |
|  |  |  | Fusobacteriaceae | -0.16 | 0.53 |
|  |  |  | Lachnospiraceae | 0.14 | 0.37 |
|  |  |  | Lactobacillaceae | -0.32 | 2.08 |
|  |  |  | Muribaculaceae | 0.85 | 14.35 |
|  |  |  | Peptostreptococcaceae | -0.89 | 15.67 |
|  |  |  | Prevotellaceae | 0.15 | 0.42 |
|  |  |  | Rhodospirillales (uncultured) | -0.8 | 2.84 |
|  |  |  | Rikenellaceae | -0.64 | 8.27 |
|  |  |  | Oscillospiraceae | 0.49 | 4.88 |
|  |  |  | Tannerellaceae | -0.78 | 12.27 |
|  |  |  | Veillonellaceae | -0.61 | 7.33 |
| PC_Class2 | 2.69 | 0.16 | Akkermansiaceae | 0.81 | 24.35 |
|  |  |  | Bacteroidaceae | -0.19 | 1.28 |
|  |  |  | Clostridiales vadinBB60 group | 0.60 | 13.27 |
|  |  |  | Enterobacteriaceae | 0.50 | 9.26 |
|  |  |  | Erysipelotrichaceae | -0.20 | 1.49 |
|  |  |  | Eubacteriaceae | -0.11 | 0.44 |
|  |  |  | Fusobacteriaceae | 0.86 | 27.43 |
|  |  |  | Lachnospiraceae | -0.19 | 1.40 |
|  |  |  | Lactobacillaceae | 0.24 | 2.10 |
|  |  |  | Muribaculaceae | -0.22 | 1.72 |
|  |  |  | Peptostreptococcaceae | 0.02 | 0.01 |
|  |  |  | Prevotellaceae | -0.01 | 0.00 |
|  |  |  | Rhodospirillales (uncultured) | -0.20 | 1.51 |
|  |  |  | Rikenellaceae | -0.32 | 3.82 |
|  |  |  | Oscillospiraceae | -0.32 | 3.89 |
|  |  |  | Tannerellaceae | -0.44 | 7.07 |
|  |  |  | Veillonellaceae | -0.16 | 0.96 |
| PC_Class3 | 1.66 | 0.10 | Akkermansiaceae | -0.02 | 0.03 |
|  |  |  | Bacteroidaceae | -0.06 | 0.22 |
|  |  |  | Clostridiales vadinBB60 group | -0.21 | 2.67 |
|  |  |  | Enterobacteriaceae | -0.27 | 4.45 |
|  |  |  | Erysipelotrichaceae | -0.33 | 6.23 |
|  |  |  | Eubacteriaceae | -0.02 | 0.02 |
|  |  |  | Fusobacteriaceae | -0.05 | 0.13 |
|  |  |  | Lachnospiraceae | -0.71 | 30.70 |
|  |  |  | Lactobacillaceae | 0.32 | 6.29 |
|  |  |  | Muribaculaceae | 0.39 | 9.00 |
|  |  |  | Peptostreptococcaceae | -0.17 | 1.800 |
|  |  |  | Prevotellaceae | 0.62 | 23.16 |
|  |  |  | Rhodospirillales (uncultured) | 0.30 | 5.54 |
|  |  |  | Rikenellaceae | -0.21 | 2.65 |
|  |  |  | Oscillospiraceae | -0.32 | 6.29 |
|  |  |  | Tannerellaceae | -0.04 | 0.09 |
|  |  |  | Veillonellaceae | 0.08 | 0.34 |
| PC_Class4 | 1.36 | 0.08 | Akkermansiaceae | 0.16 | 1.98 |
|  |  |  | Bacteroidaceae | 0.19 | 2.73 |
|  |  |  | Clostridiales vadinBB60 group | -0.38 | 10.49 |
|  |  |  | Enterobacteriaceae | 0.34 | 8.42 |
|  |  |  | Erysipelotrichaceae | 0.00 | 0.00 |
|  |  |  | Eubacteriaceae | -0.12 | 1.13 |
|  |  |  | Fusobacteriaceae | -0.24 | 4.29 |
|  |  |  | Lachnospiraceae | -0.14 | 1.54 |
|  |  |  | Lactobacillaceae | 0.01 | 0.01 |
|  |  |  | Muribaculaceae | -0.04 | 0.10 |
|  |  |  | Peptostreptococcaceae | 0.20 | 2.87 |
|  |  |  | Prevotellaceae | 0.30 | 6.71 |
|  |  |  | Rhodospirillales (uncultured) | -0.55 | 22.27 |
|  |  |  | Rikenellaceae | -0.24 | 4.41 |
|  |  |  | Oscillospiraceae | 0.47 | 16.58 |
|  |  |  | Tannerellaceae | -0.31 | 7.15 |
|  |  |  | Veillonellaceae | 0.36 | 9.33 |
| PC_Class5 | 1.18 | 0.07 | Akkermansiaceae | -0.12 | 1.31 |
|  |  |  | Bacteroidaceae | 0.33 | 9.51 |
|  |  |  | Clostridiales vadinBB60 group | -0.27 | 6.03 |
|  |  |  | Enterobacteriaceae | 0.54 | 25.06 |
|  |  |  | Erysipelotrichaceae | -0.43 | 15.71 |
|  |  |  | Eubacteriaceae | -0.24 | 4.72 |
|  |  |  | Fusobacteriaceae | -0.19 | 3.14 |
|  |  |  | Lachnospiraceae | 0.29 | 6.98 |
|  |  |  | Lactobacillaceae | 0.21 | 3.61 |
|  |  |  | Muribaculaceae | 0.02 | 0.04 |
|  |  |  | Peptostreptococcaceae | -0.04 | 0.15 |
|  |  |  | Prevotellaceae | 0.01 | 0.00 |
|  |  |  | Rhodospirillales (uncultured) | 0.27 | 6.22 |
|  |  |  | Rikenellaceae | -0.10 | 0.85 |
|  |  |  | Oscillospiraceae | 0.12 | 1.26 |
|  |  |  | Tannerellaceae | 0.03 | 0.09 |
|  |  |  | Veillonellaceae | -0.42 | 15.30 |
| PC_Class6 | 1.04 | 0.06 | Akkermansiaceae | -0.27 | 6.83 |
|  |  |  | Bacteroidaceae | -0.08 | 0.55 |
|  |  |  | Clostridiales vadinBB60 group | 0.04 | 0.12 |
|  |  |  | Enterobacteriaceae | -0.10 | 1.01 |
|  |  |  | Erysipelotrichaceae | 0.13 | 1.61 |
|  |  |  | Eubacteriaceae | 0.05 | 0.20 |
|  |  |  | Fusobacteriaceae | -0.02 | 0.05 |
|  |  |  | Lachnospiraceae | 0.01 | 0.01 |
|  |  |  | Lactobacillaceae | 0.80 | 61.21 |
|  |  |  | Muribaculaceae | 0.02 | 0.03 |
|  |  |  | Peptostreptococcaceae | 0.11 | 1.06 |
|  |  |  | Prevotellaceae | -0.23 | 5.02 |
|  |  |  | Rhodospirillales (uncultured) | -0.43 | 17.75 |
|  |  |  | Rikenellaceae | -0.10 | 1.06 |
|  |  |  | Oscillospiraceae | -0.10 | 1.02 |
|  |  |  | Tannerellaceae | 0.15 | 2.25 |
|  |  |  | Veillonellaceae | -0.05 | 0.21 |
