# Supplementary material for: The effects of antibiotics and illness on gut microbial composition in the fawn-footed mosaic-tailed rat (Melomys cervinipes)
Source: PLoS One. 2023 Feb 24;18(2):e0281533. doi: 10.1371/journal.pone.0281533 (PMC9956021; doi:10.1371/journal.pone.0281533)
Supplement: S3 Table — Generated for the various principal components analyses. Significant correlations indicated in bold. (DOCX) [file pone.0281533.s003.docx]

**Table S3.** **Spearman’s rank correlation matrices**. Generated for the various principal components analyses. Significant correlations indicated in bold.

| ***Phylum*** | Bacteroidota | Bacillota | Cyanobacteria | Pseudomonadota | Verrucomicrobiota | Fusobacteriota |
| --- | --- | --- | --- | --- | --- | --- |
| Bacteroidota | - | ***R_s_* = -0.37, *p* = 0.055** | *R_s_* = 0.05, *p* = 0.799 | *R_s_* = -0.06, *p* = 0.771 | ***R_s_* = -0.45, *p* = 0.017** | *R_s_* = -0.19, *p* = 0.322 |
| Bacillota | ***R_s_* = -0.37, *p* = 0.055** | - | *R_s_* = -0.01, *p* = 0.950 | ***R_s_* = -0.64, *p* < 0.001** | ***R_s_* = -0.38, *p* = 0.048** | ***R_s_* = -0.59, *p* < 0.001** |
| Cyanobacteria | *R_s_* = 0.05, *p* = 0.799 | *R_s_* = -0.01, *p* = 0.950 | - | *R_s_* = -0.10, *p* = 0.620 | *R_s_* = -0.30, *p* = 0.127 | *R_s_* = 0.06, *p* = 0.760 |
| Pseudomonadota | *R_s_* = -0.06, *p* = 0.771 | ***R_s_* = -0.64, *p* < 0.001** | *R_s_* = -0.10, *p* = 0.620 | - | ***R_s_* = 0.39, *p* = 0.038** | ***R_s_* = 0.56, *p* = 0.002** |
| Verrucomicrobiota | ***R_s_* = -0.45, *p* = 0.017** | ***R_s_* = -0.38, *p* = 0.048** | *R_s_* = -0.30, *p* = 0.127 | ***R_s_* = 0.39, *p* = 0.038** | - | ***R_s_* = 0.69, *p* < 0.001** |
| Fusobacteriota | *R_s_* = -0.19, *p* = 0.322 | ***R_s_* = -0.59, *p* < 0.001** | *R_s_* = 0.06, *p* = 0.760 | ***R_s_* = 0.56, *p* = 0.002** | ***R_s_* = 0.69, *p* < 0.001** | - |

| ***Class*** | Bacteroidia | Bacilli | Clostridia | Negativicutes | Fusobacteriia | Alphaproteobacteria | Gammaproteobacteria | Verrucomicrobiae |
| --- | --- | --- | --- | --- | --- | --- | --- | --- |
| Bacteroidia | - | ***R_s_* = -0.39,**  ***p* = 0.041** | *R_s_* = -0.25,  *p* = 0.203 | *R_s_* = 0.28,  *p* = 0.154 | *R_s_* = -0.19,  *p* = 0.322 | ***R_s_* = 0.46,**  ***p* = 0.015** | *R_s_* = -0.13,  *p* = 0.520 | *R_s_* = -0.45,  *p* = 0.017 |
| Bacilli | ***R_s_* = -0.39,**  ***p* = 0.041** | - | ***R_s_* = 0.37,**  ***p* = 0.049** | ***R_s_* = -0.61,**  ***p* = 0.001** | ***R_s_* = -0.40,**  ***p* = 0.033** | *R_s_* = -0.28,  *p* = 0.149 | *R_s_* = -0.35,  *p* = 0.071 | *R_s_* = -0.27,  *p* = 0.246 |
| Clostridia | *R_s_* = -0.25,  *p* = 0.203 | ***R_s_* = 0.37,**  ***p* = 0.049** | - | ***R_s_* = -0.64,**  ***p* < 0.001** | ***R_s_* = -0.55,**  ***p* = 0.003** | *R_s_* = -0.05,  *p* = 0.783 | *R_s_* = -0.37,  *p* = 0.056 | *R_s_* = -0.28,  *p* = 0.149 |
| Negativicutes | *R_s_* = 0.28,  *p* = 0.154 | ***R_s_* = -0.61,**  ***p* = 0.001** | ***R_s_* = -0.64,**  ***p* < 0.001** | - | ***R_s_* = 0.40,**  ***p* = 0.033** | *R_s_* = 0.11,  *p* = 0.566 | *R_s_* = 0.14,  *p* = 0.471 | *R_s_* = 0.15,  *p* = 0.456 |
| Fusobacteriia | *R_s_* = -0.19,  *p* = 0.322 | ***R_s_* = -0.40,**  ***p* = 0.033** | ***R_s_* = -0.55,**  ***p* = 0.003** | ***R_s_* = 0.40,**  ***p* = 0.033** | - | *R_s_* = 0.02,  *p* = 0.937 | ***R_s_* = 0.47,**  ***p* = 0.012** | ***R_s_* = 0.69,**  ***p* < 0.001** |
| Alphaproteo-  bacteria | ***R_s_* = 0.46,**  ***p* = 0.015** | *R_s_* = -0.28,  *p* = 0.149 | *R_s_* = -0.05,  *p* = 0.783 | *R_s_* = 0.11,  *p* = 0.566 | *R_s_* = 0.02,  *p* = 0.937 | - | *R_s_* = 0.01,  *p* = 0.955 | *R_s_* = -0.31,  *p* = 0.105 |
| Gammaproteo-bacteria | *R_s_* = -0.13,  *p* = 0.520 | *R_s_* = -0.35,  *p* = 0.071 | *R_s_* = -0.37,  *p* = 0.056 | *R_s_* = 0.14,  *p* = 0.471 | ***R_s_* = 0.47,**  ***p* = 0.012** | *R_s_* = 0.01,  *p* = 0.955 | - | ***R_s_* = 0.39,**  ***p* = 0.040** |
| Verruco-microbiae | *R_s_* = -0.45,  *p* = 0.017 | *R_s_* = -0.27,  *p* = 0.246 | *R_s_* = -0.28,  *p* = 0.149 | *R_s_* = 0.15,  *p* = 0.456 | ***R_s_* = 0.69,**  ***p* < 0.001** | *R_s_* = -0.31,  *p* = 0.105 | ***R_s_* = 0.39,**  ***p* = 0.040** | ***-*** |

| ***Order*** | Bacteroidales | Gastranaerophilales | Lactobacillales | Eubacteriales | Erysipelotrichales | Selenomonadales | Fusobacteriales | Rhodospirillales | Enterobacterales | Verrucomicrobiales |
| --- | --- | --- | --- | --- | --- | --- | --- | --- | --- | --- |
| Bacteroidales | - | *R_s_* = -0.05,  *p* = 0.819 | *R_s_* = -0.06,  *p* = 0.776 | *R_s_* = 0.06,  *p* = 0.781 | *R_s_* = -0.36,  *p* = 0.061 | *R_s_* = 0.30,  *p* = 0.122 | *R_s_* = -0.18,  *p* = 0.353 | ***R_s_* = 0.45,**  ***p* = 0.016** | *R_s_* = -0.31,  *p* = 0.111 | ***R_s_* = -0.43,**  ***p* = 0.023** |
| Gastranaerophilales | *R_s_* = -0.05,  *p* = 0.819 | - | *R_s_* = 0.04,  *p* = 0.850 | *R_s_* = -0.24,  *p* = 0.212 | ***R_s_* = 0.38,**  ***p* = 0.046** | *R_s_* = 0.02,  *p* = 0.911 | *R_s_* = -0.24,  *p* = 0.224 | *R_s_* = -0.06,  *p* = 0.764 | ***R_s_* = -0.49,**  ***p* = 0.009** | ***R_s_* = -0.47,**  ***p* = 0.012** |
| Lactobacillales | *R_s_* = -0.06,  *p* = 0.776 | *R_s_* = 0.04,  *p* = 0.850 | - | ***R_s_* = 0.62,**  ***p* < 0.001** | *R_s_* = -0.25,  *p* = 0.193 | *R_s_* = 0.32,  *p* = 0.101 | *R_s_* = 0.22,  *p* = 0.268 | *R_s_* = -0.29,  *p* = 0.131 | *R_s_* = 0.29,  *p* = 0.141 | *R_s_* = 0.06,  *p* = 0.744 |
| Eubacteriales | *R_s_* = 0.06,  *p* = 0.781 | *R_s_* = 0.04,  *p* = 0.850 | ***R_s_* = 0.62,**  ***p* < 0.001** | - | *R_s_* = -0.35,  *p* = 0.066 | ***R_s_* = 0.38,**  ***p* = 0.045** | *R_s_* = 0.13,  *p* = 0.521 | *R_s_* = -0.13,  *p* = 0.515 | *R_s_* = 0.35,  *p* = 0.070 | *R_s_* = -0.05,  *p* = 0.816 |
| Erysipelotrichales | *R_s_* = -0.36,  *p* = 0.061 | ***R_s_* = 0.38,**  ***p* = 0.046** | *R_s_* = -0.25,  *p* = 0.193 | *R_s_* = -0.35,  *p* = 0.066 | - | ***R_s_* = -0.47,**  ***p* = 0.011** | ***R_s_* = -0.47,**  ***p* = 0.012** | *R_s_* = -0.35,  *p* = 0.066 | ***R_s_* = -0.47,**  ***p* = 0.012** | ***R_s_* = -0.39,**  ***p* = 0.040** |
| Selenomonadales | *R_s_* = 0.30,  *p* = 0.122 | *R_s_* = 0.02,  *p* = 0.911 | *R_s_* = 0.32,  *p* = 0.101 | ***R_s_* = 0.38,**  ***p* = 0.045** | ***R_s_* = -0.47,**  ***p* = 0.011** | - | ***R_s_* = 0.40,**  ***p* = 0.033** | *R_s_* = 0.04,  *p* = 0.822 | *R_s_* = 0.35,  *p* = 0.067 | *R_s_* = 0.15,  *p* = 0.456 |
| Fusobacteriales | *R_s_* = -0.18,  *p* = 0.353 | *R_s_* = -0.24,  *p* = 0.224 | *R_s_* = 0.22,  *p* = 0.268 | *R_s_* = 0.13,  *p* = 0.521 | ***R_s_* = -0.47,**  ***p* = 0.012** | ***R_s_* = 0.40,**  ***p* = 0.033** | - | *R_s_* = 0.09,  *p* = 0.654 | ***R_s_* = 0.66,**  ***p* < 0.001** | ***R_s_* = 0.69,**  ***p* < 0.001** |
| Rhodospirillales | ***R_s_* = 0.45,**  ***p* = 0.016** | *R_s_* = -0.06,  *p* = 0.764 | *R_s_* = -0.29,  *p* = 0.131 | *R_s_* = -0.13,  *p* = 0.515 | *R_s_* = -0.35,  *p* = 0.066 | *R_s_* = 0.04,  *p* = 0.822 | *R_s_* = 0.09,  *p* = 0.654 | ***-*** | *R_s_* = -0.20,  *p* = 0.302 | *R_s_* = -0.20,  *p* = 0.297 |
| Enterobacterales | *R_s_* = -0.31,  *p* = 0.111 | ***R_s_* = -0.49,**  ***p* = 0.009** | *R_s_* = 0.29,  *p* = 0.141 | *R_s_* = 0.35,  *p* = 0.070 | ***R_s_* = -0.47,**  ***p* = 0.012** | *R_s_* = 0.35,  *p* = 0.067 | ***R_s_* = 0.66,**  ***p* < 0.001** | *R_s_* = -0.20,  *p* = 0.302 | ***-*** | ***R_s_* = 0.69,**  ***p* < 0.001** |
| Verrucomicrobiales | ***R_s_* = -0.43,**  ***p* = 0.023** | ***R_s_* = -0.47,**  ***p* = 0.012** | *R_s_* = 0.06,  *p* = 0.744 | *R_s_* = -0.05,  *p* = 0.816 | ***R_s_* = -0.39,**  ***p* = 0.040** | *R_s_* = 0.15,  *p* = 0.456 | ***R_s_* = 0.69,**  ***p* < 0.001** | *R_s_* = -0.20,  *p* = 0.297 | ***R_s_* = 0.69,**  ***p* < 0.001** | ***-*** |

| ***Class*** | Akkermansiaceae | Bacteroidaceae | Clostridiales vadin BB60 group | Enterobacteriaceae | Erysipelotrichaceae | Eubacteriaceae | Fusobacteriaceae | Lachnospiraceae | Lactobacillaceae | Muribaculaceae | Peptostreptococcaceae | Prevotellaceae | Rhodospirillales (uncultured) | Rikenellaceae | Oscillospiraceae | Tannerellaceae | Veillonellaceae |
| --- | --- | --- | --- | --- | --- | --- | --- | --- | --- | --- | --- | --- | --- | --- | --- | --- | --- |
| Akkermansiaceae | - | *R_s_* = 0.07,  *p* = 0.714 | *R_s_* = -0.01,  *p* = 0.960 | ***R_s_* = 0.69,**  ***p* < 0.001** | ***R_s_* = -0.39,**  ***p* = 0.040** | *R_s_* = -0.12,  *p* = 0.528 | ***R_s_* = 0.69,**  ***p* < 0.001** | *R_s_* = -0.27,  *p* = 0.160 | *R_s_* = 0.06,  *p* = 0.753 | *R_s_* = -0.15,  *p* = 0.432 | *R_s_* = 0.12,  *p* = 0.550 | *R_s_* = -0.04,  *p* = 0.829 | *R_s_* = -0.20,  *p* = 0.297 | *R_s_* = -0.13,  *p* = 0.508 | *R_s_* = -0.06,  *p* = 0.766 | *R_s_* = -0.36,  *p* = 0.059 | *R_s_* = -0.02,  *p* = 0.933 |
| Bacteroidaceae | *R_s_* = 0.07,  *p* = 0.714 | - | ***R_s_* = -058,**  ***p* = 0.001** | *R_s_* = 0.08,  *p* = 0.694 | *R_s_* = -0.36,  *p* = 0.057 | *R_s_* = -0.36,  *p* = 0.060 | *R_s_* = 0.25,  *p* = 0.203 | *R_s_* = -0.24,  *p* = 0.214 | *R_s_* = 0.27,  *p* = 0.161 | ***R_s_* = -0.43,**  ***p* = 0.023** | ***R_s_* = 0.47,**  ***p* = 0.012** | *R_s_* = -0.09,  *p* = 0.633 | *R_s_* = 0.12,  *p* = 0.541 | ***R_s_* = 0.51,**  ***p* = 0.006** | ***R_s_* = -0.38,**  ***p* = 0.049** | ***R_s_* = 0.60,**  ***p* < 0.001** | ***R_s_* = 0.71,**  ***p* < 0.001** |
| Clostridiales vadin BB60 group | *R_s_* = -0.01,  *p* = 0.960 | ***R_s_* = -058,**  ***p* = 0.001** | - | ***R_s_* = -0.43,**  ***p* = 0.021** | ***R_s_* = 0.50,**  ***p* = 0.006** | ***R_s_* = 0.72,**  ***p* < 0.001** | *R_s_* = -0.25,  *p* = 0.196 | *R_s_* = 0.24,  *p* = 0.222 | ***R_s_* = -0.57,**  ***p* = 0.002** | ***R_s_* = 0.62,**  ***p* < 0.001** | ***R_s_* = -0.79,**  ***p* < 0.001** | *R_s_* = 0.21,  *p* = 0.283 | *R_s_* = -0.08,  *p* = 0.689 | ***R_s_* = -0.50,**  ***p* = 0.007** | *R_s_* = 0.30,  *p* = 0.120 | ***R_s_* = -0.56,**  ***p* = 0.002** | ***R_s_* = -0.66,**  ***p* < 0.001** |
| Enterobacteriaceae | ***R_s_* = 0.69,**  ***p* < 0.001** | *R_s_* = 0.08,  *p* = 0.694 | ***R_s_* = -0.43,**  ***p* = 0.021** | - | ***R_s_* = -0.47,**  ***p* = 0.012** | ***R_s_* = -0.49,**  ***p* = 0.009** | ***R_s_* = 0.66,**  ***p* < 0.001** | *R_s_* = -0.27,  *p* = 0.161 | *R_s_* = 0.37,  *p* = 0.055 | ***R_s_* = -0.58,**  ***p* = 0.001** | ***R_s_* = 0.61,**  ***p* < 0.001** | *R_s_* = -0.14,  *p* = 0.476 | *R_s_* = -0.20,  *p* = 0.302 | *R_s_* = 0.22,  *p* = 0.266 | *R_s_* = -0.26,  *p* = 0.190 | *R_s_* = -0.04,  *p* = 0.838 | *R_s_* = 0.17,  *p* = 0.377 |
| Erysipelotrichaceae | ***R_s_* = -0.39,**  ***p* = 0.040** | *R_s_* = -0.36,  *p* = 0.057 | ***R_s_* = 0.50,**  ***p* = 0.006** | ***R_s_* = -0.47,**  ***p* = 0.012** | - | ***R_s_* = 0.41,**  ***p* = 0.032** | ***R_s_* = -0.46,**  ***p* = 0.014** | *R_s_* = 0.23,  *p* = 0.239 | *R_s_* = -0.35,  *p* = 0.071 | *R_s_* = 0.32,  *p* = 0.097 | ***R_s_* = -0.46,**  ***p* = 0.014** | *R_s_* = -0.09,  *p* = 0.654 | *R_s_* = -0.34,  *p* = 0.077 | *R_s_* = -0.05,  *p* = 0.808 | ***R_s_* = 0.42,**  ***p* = 0.025** | *R_s_* = -0.29,  *p* = 0.132 | *R_s_* = -0.32,  *p* = 0.097 |
| Eubacteriaceae | *R_s_* = -0.12,  *p* = 0.528 | *R_s_* = -0.36,  *p* = 0.060 | ***R_s_* = 0.72,**  ***p* < 0.001** | ***R_s_* = -0.49,**  ***p* = 0.009** | ***R_s_* = 0.41,**  ***p* = 0.032** | - | ***R_s_* = -0.41,**  ***p* = 0.030** | *R_s_* = 0.17,  *p* = 0.379 | ***R_s_* = -0.76,**  ***p* < 0.001** | ***R_s_* = 0.82,**  ***p* < 0.001** | ***R_s_* = -0.84,**  ***p* < 0.001** | *R_s_* = 0.27,  *p* = 0.162 | *R_s_* = 0.06,  *p* = 0.760 | *R_s_* = -0.29,  *p* = 0.140 | ***R_s_* = 0.58,**  ***p* = 0.001** | ***R_s_* = -0.50,**  ***p* = 0.007** | ***R_s_* = -0.62,**  ***p* < 0.001** |
| Fusobacteriaceae | ***R_s_* = 0.69,**  ***p* < 0.001** | *R_s_* = 0.25,  *p* = 0.203 | *R_s_* = -0.25,  *p* = 0.196 | ***R_s_* = 0.66,**  ***p* < 0.001** | ***R_s_* = -0.46,**  ***p* = 0.014** | ***R_s_* = -0.41,**  ***p* = 0.030** | - | ***R_s_* = -0.49,**  ***p* = 0.009** | *R_s_* = 0.25,  *p* = 0.204 | *R_s_* = -0.36,  *p* = 0.060 | *R_s_* = 0.36,  *p* = 0.057 | *R_s_* = -0.08,  *p* = 0.679 | *R_s_* = 0.09,  *p* = 0.654 | *R_s_* = 0.28,  *p* = 0.146 | ***R_s_* = -0.42,**  ***p* = 0.025** | *R_s_* = -0.04,  *p* = 0.837 | *R_s_* = 0.29,  *p* = 0.137 |
| Lachnospiraceae | *R_s_* = -0.27,  *p* = 0.160 | *R_s_* = -0.24,  *p* = 0.214 | *R_s_* = 0.24,  *p* = 0.222 | *R_s_* = -0.27,  *p* = 0.161 | *R_s_* = 0.23,  *p* = 0.239 | *R_s_* = 0.17,  *p* = 0.379 | ***R_s_* = -0.49,**  ***p* = 0.009** | - | *R_s_* = -0.18,  *p* = 0.359 | *R_s_* = -0.05,  *p* = 0.799 | *R_s_* = -0.16,  *p* = 0.422 | *R_s_* = -0.01,  *p* = 0.965 | *R_s_* = 0.03,  *p* = 0.888 | *R_s_* = -0.03,  *p* = 0.862 | *R_s_* = 0.37,  *p* = 0.051 | *R_s_* = -0.10,  *p* = 0.622 | *R_s_* = -0.21,  *p* = 0.284 |
| Lactobacillaceae | *R_s_* = 0.06,  *p* = 0.753 | *R_s_* = 0.27,  *p* = 0.161 | ***R_s_* = -0.57,**  ***p* = 0.002** | *R_s_* = 0.37,  *p* = 0.055 | *R_s_* = -0.35,  *p* = 0.071 | ***R_s_* = -0.76,**  ***p* < 0.001** | *R_s_* = 0.25,  *p* = 0.204 | *R_s_* = -0.18,  *p* = 0.359 | - | ***R_s_* = -0.66,**  ***p* < 0.001** | ***R_s_* = 0.76,**  ***p* < 0.001** | *R_s_* = -0.10,  *p* = 0.628 | *R_s_* = -0.22,  *p* 0.256 | *R_s_* = 0.07,  *p* = 0.729 | ***R_s_* = -0.43,**  ***p* = 0.023** | *R_s_* = 0.37,  *p* = 0.055 | ***R_s_* = 0.54,**  ***p* = 0.003** |
| Muribaculaceae | *R_s_* = -0.15,  *p* = 0.432 | ***R_s_* = -0.43,**  ***p* = 0.023** | ***R_s_* = 0.62,**  ***p* < 0.001** | ***R_s_* = -0.58,**  ***p* = 0.001** | *R_s_* = 0.32,  *p* = 0.097 | ***R_s_* = 0.82,**  ***p* < 0.001** | *R_s_* = -0.36,  *p* = 0.060 | *R_s_* = -0.05,  *p* = 0.799 | ***R_s_* = -0.66,**  ***p* < 0.001** | - | ***R_s_* = -0.89,**  ***p* < 0.001** | *R_s_* = 0.30,  *p* = 0.125 | *R_s_* = 0.06,  *p* = 0.777 | ***R_s_* = -0.45,**  ***p* = 0.016** | ***R_s_* = 0.52,**  ***p* = 0.005** | ***R_s_* = -0.51,**  ***p* = 0.005** | ***R_s_* = -0.51,**  ***p* = 0.005** |
| ***R_s_* = -0.51,**  ***p* = 0.005** | *R_s_* = 0.12,  *p* = 0.550 | ***R_s_* = 0.47,**  ***p* = 0.012** | ***R_s_* = -0.79,**  ***p* < 0.001** | ***R_s_* = 0.61,**  ***p* < 0.001** | ***R_s_* = -0.46,**  ***p* = 0.014** | ***R_s_* = -0.84,**  ***p* < 0.001** | *R_s_* = 0.36,  *p* = 0.057 | *R_s_* = -0.16,  *p* = 0.422 | ***R_s_* = 0.76,**  ***p* < 0.001** | ***R_s_* = -0.89,**  ***p* < 0.001** | - | *R_s_* = -0.26,  *p* = 0.174 | *R_s_* = -0.11,  *p* = 0.591 | ***R_s_* = 0.48,**  ***p* = 0.009** | ***R_s_* = -0.55,**  ***p* = 0.003** | ***R_s_* = 0.59,**  ***p* = 0.001** | ***R_s_* = 0.61,**  ***p* < 0.001** |
| Prevotellaceae | *R_s_* = -0.04,  *p* = 0.829 | *R_s_* = -0.09,  *p* = 0.633 | *R_s_* = 0.21,  *p* = 0.283 | *R_s_* = -0.14,  *p* = 0.476 | *R_s_* = -0.09,  *p* = 0.654 | *R_s_* = 0.27,  *p* = 0.162 | *R_s_* = -0.08,  *p* = 0.679 | *R_s_* = -0.01,  *p* = 0.965 | *R_s_* = -0.10,  *p* = 0.628 | *R_s_* = 0.30,  *p* = 0.125 | *R_s_* = -0.26,  *p* = 0.174 | - | *R_s_* = 0.34,  *p* = 0.073 | *R_s_* = -0.08,  *p* = 0.670 | *R_s_* = 0.07,  *p* = 0.721 | *R_s_* = -0.01,  *p* = 0.968 | *R_s_* = -0.15,  *p* = 0.446 |
| Rhodospirillales (uncultured) | *R_s_* = -0.20,  *p* = 0.297 | *R_s_* = 0.12,  *p* = 0.541 | *R_s_* = -0.08,  *p* = 0.689 | *R_s_* = -0.20,  *p* = 0.302 | *R_s_* = -0.34,  *p* = 0.077 | *R_s_* = 0.06,  *p* = 0.760 | *R_s_* = 0.09,  *p* = 0.654 | *R_s_* = 0.03,  *p* = 0.888 | *R_s_* = -0.22,  *p* 0.256 | *R_s_* = 0.06,  *p* = 0.777 | *R_s_* = -0.11,  *p* = 0.591 | *R_s_* = 0.34,  *p* = 0.073 | - | *R_s_* = 0.25,  *p* = 0.203 | *R_s_* = -0.31,  *p* = 0.110 | *R_s_* = 0.35,  *p* = 0.067 | *R_s_* = 0.00,  *p* = 0.999 |
| Rikenellaceae | *R_s_* = -0.13,  *p* = 0.508 | ***R_s_* = 0.51,**  ***p* = 0.006** | ***R_s_* = -0.50,**  ***p* = 0.007** | *R_s_* = 0.22,  *p* = 0.266 | *R_s_* = -0.05,  *p* = 0.808 | *R_s_* = -0.29,  *p* = 0.140 | *R_s_* = 0.28,  *p* = 0.146 | *R_s_* = -0.03,  *p* = 0.862 | *R_s_* = 0.07,  *p* = 0.729 | ***R_s_* = -0.45,**  ***p* = 0.016** | ***R_s_* = 0.48,**  ***p* = 0.009** | *R_s_* = -0.08,  *p* = 0.670 | *R_s_* = 0.25,  *p* = 0.203 | - | *R_s_* = -0.19,  *p* = 0.327 | ***R_s_* = 0.54,**  ***p* = 0.003** | ***R_s_* = 0.46,**  ***p* = 0.014** |
| Oscillospiraceae | *R_s_* = -0.06,  *p* = 0.766 | ***R_s_* = -0.38,**  ***p* = 0.049** | *R_s_* = 0.30,  *p* = 0.120 | *R_s_* = -0.26,  *p* = 0.190 | ***R_s_* = 0.42,**  ***p* = 0.025** | ***R_s_* = 0.58,**  ***p* = 0.001** | ***R_s_* = -0.42,**  ***p* = 0.025** | *R_s_* = 0.37,  *p* = 0.051 | ***R_s_* = -0.43,**  ***p* = 0.023** | ***R_s_* = 0.52,**  ***p* = 0.005** | ***R_s_* = -0.55,**  ***p* = 0.003** | *R_s_* = 0.07,  *p* = 0.721 | *R_s_* = -0.31,  *p* = 0.110 | *R_s_* = -0.19,  *p* = 0.327 | - | ***R_s_* = -0.57**  ***p* = 0.002** | ***R_s_* = -0.41,**  ***p* = 0.031** |
| Tannerellaceae | *R_s_* = -0.36,  *p* = 0.059 | ***R_s_* = 0.60,**  ***p* < 0.001** | ***R_s_* = -0.56,**  ***p* = 0.002** | *R_s_* = -0.04,  *p* = 0.838 | *R_s_* = -0.29,  *p* = 0.132 | ***R_s_* = -0.50,**  ***p* = 0.007** | *R_s_* = -0.04,  *p* = 0.837 | *R_s_* = -0.10,  *p* = 0.622 | *R_s_* = 0.37,  *p* = 0.055 | ***R_s_* = -0.51,**  ***p* = 0.005** | ***R_s_* = 0.59,**  ***p* = 0.001** | *R_s_* = -0.01,  *p* = 0.968 | *R_s_* = 0.35,  *p* = 0.067 | ***R_s_* = 0.54,**  ***p* = 0.003** | ***R_s_* = -0.57**  ***p* = 0.002** | - | ***R_s_* = 0.53,**  ***p* = 0.004** |
| Veillonellaceae | *R_s_* = -0.02,  *p* = 0.933 | ***R_s_* = 0.71,**  ***p* < 0.001** | ***R_s_* = -0.66,**  ***p* < 0.001** | *R_s_* = 0.17,  *p* = 0.377 | *R_s_* = -0.32,  *p* = 0.097 | ***R_s_* = -0.62,**  ***p* < 0.001** | *R_s_* = 0.29,  *p* = 0.137 | *R_s_* = -0.21,  *p* = 0.284 | ***R_s_* = 0.54,**  ***p* = 0.003** | ***R_s_* = -0.51,**  ***p* = 0.005** | ***R_s_* = 0.61,**  ***p* < 0.001** | *R_s_* = -0.15,  *p* = 0.446 | *R_s_* = 0.00,  *p* = 0.999 | ***R_s_* = 0.46,**  ***p* = 0.014** | ***R_s_* = -0.41,**  ***p* = 0.031** | ***R_s_* = 0.53,**  ***p* = 0.004** | - |
